# Misdiagnosis of eosinophilic cystitis: A case report and literature review

**DOI:** 10.1097/MD.0000000000036668

**Published:** 2024-02-16

**Authors:** Guanyu Shi, Leibo Wang, Guangxu Peng, Xu An, Xingyong Lu, Huagu Wu, Yongjun Li

**Affiliations:** a Department of Urology, Fenggang County People’s Hospital, Fenggang, Guizhou, China; b Department of Surgery, Guizhou Orthopaedic Hospital, Guiyang, Guizhou, China.

**Keywords:** diagnosis, eosinophilic cystitis, misdiagnosis, treatment

## Abstract

**Rationale:**

Eosinophilic cystitis (EC) is a rare and specific transmural inflammatory disease in clinic. At present, its etiology is unknown, its clinical manifestations are diverse, and its auxiliary examination lacks specificity, so it is easy to be missed or misdiagnosed in clinical practice.

**Patient concerns:**

A 72-year-old male patient with symptoms of lower urinary tract obstruction accompanied by hematuria was diagnosed with benign prostatic hyperplasia with bleeding by B-ultrasound and urinary CT examination. After being treated with catheterization, anti-infection and hemostasis, he was selectively treated with transurethral resection of prostate, but he saw a pattern mass on the right back wall of the bladder during the operation. Considering bladder tumor, he removed the lesion and gave pirarubicin for bladder perfusion. However, the postoperative pathological result was EC.

**Diagnosis:**

The diagnosis of EC can only rely on pathological examination, and the accurate and positive rate of biopsy can be improved by obtaining muscle tissue as much as possible at the same time of multi-point biopsy.

**Intervention:**

Prednisone and cetirizine were given orally after transurethral resection of lesions, and tamsulosin and finasteride were given regularly to treat benign prostatic hyperplasia.

**Outcomes:**

No recurrence and abnormal urination were found during the follow-up for half a year, and the upper urinary tract function was normal.

**Lessons:**

The clinical manifestations of EC are atypical, the laboratory examination and imaging examination are not specific, and it is difficult to make a definite diagnosis before operation. The diagnosis depends on pathological examination. Transurethral resection of the lesion can obviously improve the positive rate of biopsy while completely removing the lesion, and the combined drug treatment can achieve satisfactory results in a short period of time. Active follow-up after operation is very important to identify the recurrence of the disease and prevent the upper urinary tract function from being damaged.

## 1. Introduction

Eosinophilic cystitis (EC) is a rare inflammatory disease of bladder. At present, the etiology is unknown, which may be related to allergic reaction, bladder trauma, lower urinary tract obstruction, infection, local irritation of bladder, etc.^[1]^ The main clinical manifestations are urgency, dysuria, hematuria and pelvic pain.^[2]^ Similar to other common lower urinary tract diseases, it is easy to be missed or misdiagnosed. This paper reports a case of misdiagnosis of EC, aiming to remind clinicians of the importance of understanding, diagnosis and treatment of rare diseases.

## 2. Case presentation

**Figure 1.**
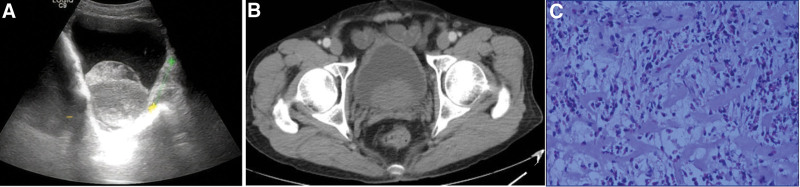
Imaging examination and pathological results of eosinophilic cystitis (A) B-ultrasound image; (B) CT image; (C) postoperative pathology (HE, ×400).

A 72-year-old male patient was admitted to the hospital because of “dysuria for 1 year, aggravated with frequent micturition, urgency and hematuria for 2 days.” One year ago, the patient suffered from dysuria, thinner urinary line, shorter range, dribbling micturition and increased nocturia, about 4 times per night, without special diagnosis and treatment, and his condition was not relieved; Two days ago, the patient felt that urination was aggravated, accompanied by frequent urination, urgency and gross hematuria. No history of allergy, surgery and drug abuse. Physical examination: the bladder area is full and tender, and the rest is normal. Auxiliary examination: urine routine: nitrite+, white blood cells-, urinary protein 3+, occult blood 3+, red blood cell count 63547/µL, white blood cells 0/µL. Blood routine: the number of eosinophils is 0.15 × 10^9^/L, the percentage of eosinophils is 2.4%, the number of leukocytes is 6.14 × 10^9^/L, the percentage of neutrophils is 75.2%, and the number of platelets is 269 × 10^9^/L; The urine was cultured as Citrobacter krylovii; There was no obvious abnormality in coagulation function, liver and kidney function and prostate-specific antigen. B-ultrasound of urinary system (Fig. 1A): the bladder is over-filled, the inner wall is smooth, strong echo mass can be seen in the dark area, the range is about 67 mm × 47 mm, the position changes with the body position, the prostate is about 51 mm × 47 mm × 32 mm, the shape is full, the echo is uneven, and no obvious abnormal blood flow signal is found in CDFI; Urinary CT (Fig. 1B): the prostate is enlarged, the bladder wall is smooth, and no obvious thickening is found. Preliminary diagnosis: prostatic hyperplasia with bleeding, acute urinary retention, urinary tract infection. After admission, indwelling catheterization led to bloody urine and dark brown blood clots, which were treated with anti-infection and hemostasis. Transurethral resection of the prostate was performed selectively. During the operation, the bilateral lobes of the prostate were hyperplasia and the neck was obviously raised. A cauliflower mass with a size of about 1.0 cm × 1.5 cm was seen in the right posterior wall of the bladder, which was not flushed, no active bleeding was found, and little old blood clots were attached to the tumor surface, but no calcification and necrosis were found. Therefore, the bladder tumor was considered during the operation, and the lesion was removed. Postoperative pathology (Fig. 1C): EC, so oral prednisone and cetirizine were given, and tamsulosin and finasteride were given regularly to treat benign prostatic hyperplasia. After half a year follow-up, the patient had no abnormal urination, and no abnormalities were found in blood routine, urine routine, B-ultrasound and CT, and no congestion, edema and tumor of bladder mucosa were found in cystoscopy.

## 3. Discussion

EC is a specific transmural cystitis with eosinophil infiltration.^[3]^ Since it was first reported in 1960, only about 200 cases have been reported in the world so far.^[4]^ The literature is almost a small case study or case report. EC can occur at any age, especially in children and young women. At present, the etiology is not completely clear, and it is generally considered to be related to allergic reaction. Allergy induces lysosomes in eosinophils to degrade and release urinary eosinophil cationic protein, which can enhance bladder inflammatory reaction, and then cause bladder mucosa and muscularis fibrosis, detrusor fibrosis and muscularis necrosis, thus causing various symptoms.^[5]^ Others, such as urinary tract infection, long-term indwelling catheter, prostatic hyperplasia, surgery, trauma, bladder perfusion of chemotherapy drugs, bladder tumor, etc, can cause EC.^[6]^ Some scholars infer that the occurrence of this disease may be related to eosinophilic lesions in other systems or parts, but the cause is unknown.^[7]^ It has also been reported that it may be related to the mutation of BRAF 1463T gene.^[8,9]^ This case has no history of allergy and bladder surgery, except prostate hyperplasia and urinary tract infection.

The clinical manifestations of EC are diverse and atypical, and the common symptoms include frequent urination, urgency, dysuria, hematuria and pelvic pain, etc. When inflammation invades the bladder neck, urinary incontinence, dysuria and urinary retention may occur, and when hydronephrosis occurs, low back pain and knocking pain in the renal area may occur,^[10,11]^ so it is often confused with other inflammatory diseases, bladder tumors, prostatic hyperplasia, etc. Since the first report, the number of EC cases is small, which may be in clinical practice.

The diagnostic value of laboratory examination for EC is not high. About 43% of EC patients have eosinophils in peripheral blood > 5% of total white blood cells,^[12]^ and a few patients can have eosinophils in urine but lack specificity.^[13]^ Urine culture is mostly negative, and when combined with bacterial infection, urine white blood cell count may increase and urine culture may be positive. The specificity of imaging examination is not strong, and the main hints are irregular thickening of bladder and focal mass. Some scholars^[14]^ have found that the MRI manifestations of EC have certain characteristics, such as low signal on T2WI, limited diffusion of DWI and gradual enhancement, which have certain clinical value for disease diagnosis. Extensive mucosal erythema, mucosal hyperemia and edema, and space-occupying lesions can be seen under cystoscope,^[15]^ but it is still difficult to distinguish them from bladder tumors under cystoscope.^[16]^ Pathological examination is the gold standard for the diagnosis of EC, and pathological examination shows that a large number of eosinophils infiltrate bladder mucosa and muscularis. Ultrasound-guided biopsy is a safe and effective alternative diagnostic method for EC patients who cannot undergo cystoscopy.^[17]^

Analysis of the causes of misdiagnosis in this case: At present, there are few reports about 1. EC at home and abroad, which leads to an insufficient understanding of this disease. An elderly man came to the hospital with the following symptoms of urinary tract obstruction and considered it as benign prostatic hyperplasia with clinical inertia thinking. The clinical manifestations of this disease are diverse and nonspecific, and the diagnosis of rare diseases is ignored by the diagnosis and treatment of common and frequently-occurring diseases related to symptoms. EC was not considered before and during this case, and it is more likely to be missed or misdiagnosed when EC is combined with other clinical manifestations of urinary system similar diseases. The imaging examination of 3.EC has no specificity. Previous literature reported that the imaging manifestations were mainly bladder wall thickening or tumor-like mass. In this case, the imaging examination of bladder wall showed no obvious abnormal thickening, and it was considered that the emergency imaging examination time was hasty, the center of gravity was subjectively shifted and the equipment was defective. Bladder tumor was found during operation, and combined with hematuria history, it was satisfied that the diagnosis of bladder tumor did not undergo intraoperative pathological examination. Therefore, in the face of similar symptoms in clinical work, the possibility of rare diseases should be considered, and corresponding examinations should be carried out to exclude other diseases and avoid misdiagnosis and delayed treatment.

At present, there is no consensus on the treatment of EC. Some scholars believe that EC is a self-limiting disease,^[18]^ but it is often necessary to improve symptoms and prevent the progression of the disease as soon as possible in clinic. Therefore, once diagnosed, we should focus on suppressing immune response, reducing inflammatory damage of bladder wall and protecting upper urinary tract from secondary damage. At present, the mainstream tends to adopt conservative treatment first, remove suspected allergens or inducements, and treat children with mild symptoms for 6–8 weeks.^[19]^ It has been reported that Benalizumab, an interleukin-5 antagonist, may have a significant effect on EC, but it still needs to be further confirmed in clinical trials.^[20]^ Antibiotics can be used appropriately for patients with bacterial infection. If the patient condition is still progressing after conservative treatment, combined transurethral resection is a more effective treatment. If the patient condition is still progressing after drug treatment and transurethral resection, partial cystectomy, total cystectomy and urinary diversion should be considered.^[21]^ After transurethral resection of lesions, this case was treated with hormones and antihistamines. After active follow-up, there was no recurrence and the effect was satisfactory. At the same time, benign prostatic hyperplasia is a suspicious cause of this case. After operation, the patient was instructed to take oral drugs regularly to treat benign prostatic hyperplasia, and the urination during the follow-up period was acceptable.

To sum up, the clinical manifestations of EC are atypical, and the auxiliary examination lacks specificity. The diagnosis depends on the multi-point pathological biopsy under cystoscope, but the biopsy under cystoscope can not guarantee the acquisition of muscle tissue, while the lesion can be completely removed by transurethral resection, which greatly improves the positive rate of biopsy. At the same time, with the resection of the lesion, combined drug treatment can obviously improve the symptoms and prevent the disease from progressing in a short period of time. Therefore, the author believes that on the basis of removing suspicious causes, Transurethral resection of lesions combined with drug therapy is an effective treatment scheme for EC. Surgical trauma has little effect on bladder function, and at the same time, it can completely remove the lesions visible to the naked eye. At the same time, it can greatly improve the detection rate of this disease, and at the same time, combined with drug therapy can obtain satisfactory results in a short period of time. When there is urinary tract infection or upper urinary tract dilatation, antibiotics and other intervention measures should be used to prevent the secondary damage of upper urinary tract function. This disease may recur, and it needs more than half a year active follow-up after operation. Follow-up review is very important to identify the recurrence of this disease and prevent the upper urinary tract function from being damaged. Of course, there is still a lot of comprehensive research on the etiology and drug selection of EC to put forward a standardized consensus on diagnosis and treatment.

## Author contributions

**Conceptualization:** Guanyu Shi.

**Data curation:** Guanyu Shi, Guangxu Peng, Xu An, Xingyong Lu.

**Formal analysis:** Guanyu Shi, Guangxu Peng.

**Investigation:** Xu An, Xingyong Lu.

**Supervision:** Huagu Wu, Yongjun Li.

**Validation:** Guanyu Shi.

**Writing – original draft:** Guanyu Shi.

**Writing – review & editing:** Guanyu Shi, Leibo Wang.
